# User Experience in Social Robots

**DOI:** 10.3390/s21155052

**Published:** 2021-07-26

**Authors:** Elaheh Shahmir Shourmasti, Ricardo Colomo-Palacios, Harald Holone, Selina Demi

**Affiliations:** Department of Computer Science, Østfold University College, 1783 Halden, Norway; elahehs@hiof.no (E.S.S.); harald.holone@hiof.no (H.H.); selina.demi@hiof.no (S.D.)

**Keywords:** social robots, human-robot interaction, UX evaluation, systematic literature review

## Abstract

Social robots are increasingly penetrating our daily lives. They are used in various domains, such as healthcare, education, business, industry, and culture. However, introducing this technology for use in conventional environments is not trivial. For users to accept social robots, a positive user experience is vital, and it should be considered as a critical part of the robots’ development process. This may potentially lead to excessive use of social robots and strengthen their diffusion in society. The goal of this study is to summarize the extant literature that is focused on user experience in social robots, and to identify the challenges and benefits of UX evaluation in social robots. To achieve this goal, the authors carried out a systematic literature review that relies on PRISMA guidelines. Our findings revealed that the most common methods to evaluate UX in social robots are questionnaires and interviews. UX evaluations were found out to be beneficial in providing early feedback and consequently in handling errors at an early stage. However, despite the importance of UX in social robots, robot developers often neglect to set UX goals due to lack of knowledge or lack of time. This study emphasizes the need for robot developers to acquire the required theoretical and practical knowledge on how to perform a successful UX evaluation.

## 1. Introduction

Robots can be defined as machines that can sense and react to the events around them [1]. The creation of recent robots includes electromechanical systems that need numerous energy conversions to convert the existing energy resources to the power that is needed for these electromechanical systems [2]. Accordingly, it is necessary to develop new types of robots that can perform their functionality by mixing the attributes from living biological materials with electromechanical systems [2]. Unsurprisingly, recent technological advances provide robust solutions to a variety of technical problems that have limited robots development over years. These technological solutions led to an ever-increasing integration of robots into our physical and social settings [3]. One of the traditional strategies to achieve this integration is the humanoid form of robots. Although human-robot collaboration is an area of expansion in both research and industry [4], the ultimate quest of roboticists is to develop fully human-like robots [3]. In the future, robots are expected to possess high level of skills to fulfill humans’ expectations and assist them in any circumstances. In this regard, social robots researchers aim to develop robots with natural social interaction [5].

Duffy et al. [6] defined social robots (SR) as “a physical entity embodied in a complex, dynamic, and social environment sufficiently empowered to behave in a manner conducive to its own goals and those of its community”. The increasing need for social robots for both entertainment and education has fostered the revolution of this technology. The first works devoted to the connection between human intelligence and machines occurred in the middle of the twentieth century. Humanoid robots can grasp human mental resources by imitating their characteristics [7]. Socialization with such robots is similar to socialization with other humans. These robots are socially intelligent in a way that they can understand and respond to humans based on the situation and learn how to behave by experiencing real-life circumstances. In addition, the development of these robots has the potential to enhance the level of acknowledgment about ourselves [8]. Currently, social robots are becoming progressively common elements of our world [9,10]. Social robots are present in many areas, including education [11,12,13] and learning [14,15,16], health [17,18,19,20], care [21], tourism and hospitality [22,23,24,25,26], media [27,28], services in general [29,30], or public spaces [31,32,33,34], citing just some of the most frequent, recent, and relevant uses. Focusing on service delivery, a recent publication underlines that the adoption of social robots in such environments is motivated by branding strategies rather than functional purposes [35]. Fashion, hype or need, social robots are here to stay.

Communicating with social robots, understanding their behaviors, and developing user experience over time requires longitudinal studies, and it seems that robotics in home appliances research have been at the center of the attention [36]. Mandal [37] defined communication as conveying information through signals which are elements that are perceived by touch, sound, smell, and sight. The author stated that a signal connects the sender to the receiver and consists of three components: the signal itself, what it refers to, and the interpreter. Body language is the daily usage of this concept. We use body language to interact with people without even noticing it. Body posture and gesture, facial expression, hands, and head movements are all part of nonverbal behaviors and communications. Robots are not an exception when it comes to human-robot interaction [37]. To deliver communications, it is necessary to explain matters. Therefore, humans use their verbal and nonverbal actions to convey their defining characteristics. Likewise, social robots need this coordination to perform human-like behaviors [38]. Previous studies have focused on the trends that humans gain information or skills from each other [39]. This focus on robotics social learning can be explained by the increasing interest in developing robots that can be customized by ordinary people to be used at home, work, and public spaces [39]. The uses and capabilities of social robots are deeply analyzed in a recent literature study [40].

To successfully interact with social robots, a positive user experience (UX) is a significant matter in order to have a great impact on human life. Therefore, to fulfill this purpose, the design and evaluation of this process needs to be performed accurately [41]. As indicated by Hartson et al. [42], user experience is “the totality of the effect or effects felt by a user as a result of interaction with, and the usage context of, a system, device, or product, including the influence of usability, usefulness, and emotional impact during interaction and savoring memory after interaction”. The international standard on ergonomics of human-system interaction provides a simpler definition of UX that entails the perceptions and responses that are produced as a result of the use or anticipated use of a specific system, product or service [43]. In this study, the authors focus on the concept of UX evaluation which constitutes a set of methods and techniques that are adopted to explore how users perceive an interactive service, system or product [44]. It is worthy to note that the focus is not on evaluating users, but their experience of interacting with social robots.

User experience is remarkably crucial for a product to be successful, and extracting this information from users is not a trivial task. Some factors like psychophysiological behaviors that are very significant for measuring user experience, are not completely considered, and the product usage is not studied continuously [45]. Moreover, researchers prefer techniques such as questionnaires and interviews over real-time procedures [45]. Maia and Furtado [45] carried out a systematic literature review about user experience evaluation and found out that 84% of the studies use questionnaires and 16% of the studies used interviews to assess user experience.

Although the concept of UX is significant in the context of social robots, due to the need to communicate with robots, to the best of our knowledge, there is no previous systematic literature review on the integration between the two topics: UX and social robots. Therefore, we carried out a systematic literature review (SLR) in order to summarize the extant literature on the topic in a comprehensive and concise manner. This overview may be of interest to UX researchers and robot developers willing to consider the UX perspective during the whole social robot development process.

## 2. Research Methodology

This section introduces the research methodology adopted to search, select, and analyze previously published research that covers the topic of user experience in social robots.

### 2.1. Systematic Literature Review Using Prisma Guidelines

We carried out a systematic literature review that aims to synthesize the extant literature about user experience in social robots. This review follows the Preferred Reporting Items for Systematic Reviews and Meta-Analysis (PRISMA) guidelines [46,47,48]. This approach has been used by several high-quality papers in different fields of study, e.g., [49,50,51]. It is worth mentioning that prior to conducting the systematic literature review, authors assessed its novelty. Authors acknowledge the existence of several systematic literature reviews in the broad field of robotics [52,53,54,55,56], some of them in the field of human-robot interaction [57,58,59,60]. However, in the specific field of social robots, there are two works worth mention, one on the interaction with sexbots [61] and another on specific design guidelines for social robots for the elderly [62]. To the best of our knowledge, an SLR that focuses on user experience in social robots does not exist in the extant literature. Bearing in mind the goal of this study, the authors formulated three research questions (RQ_i_), as follows:

- RQ_1_: What has been studied about user experience in social robots operating in different domains?
- RQ_2_: What are the reported techniques for assessing UX in social robots?
- RQ_3_: What are the reported challenges and benefits in evaluating UX for social robots?

### 2.2. Eligibility Criteria

The study is eligible for review if it fits the following inclusion criteria: (i) The study is presented in the form of an article, report, white paper, thesis, or book. (ii) The study discusses the concepts of social robots and user experience. (iii) The study describes the methods of UX assessment for social robots (iv) The study identifies the challenges and benefits of UX evaluation for social robots (v) The study has been published within the last 10 years. The study is excluded if: (i) The full text version is not accessible. (ii) It is not written in English. (iii) It focuses on UX in domains other than social robots.

### 2.3. Information Sources and Search

Literature searches were conducted in five major databases:

- IEEE Xplore
- ACM Digital Library
- Springer
- ScienceDirect
- Google Scholar

The selected databases have been used broadly in secondary studies in the broad field of computing [63,64,65]. The search process aims to retrieve as many studies related to the topic of interest as possible. Bearing this in mind, two key terms were included in the search string: “user experience” and “social robot”. The term “user experience” has been also referred to as “UX”. Therefore, these keywords were included in the search string. The aforementioned search terms were structured by using the Boolean operators: “AND” for concatenation of the terms, “OR” for alternative terms.

Consequently, we formulated the following search string:

(“user experience” OR “UX”) AND “social robot”

Regarding the term “social robots”, we observed that some of the studies used the term “humanoid robot”. Therefore, we carried out trial searches with this term. However, we did not identify other relevant studies. The only filter used was to delimit the time range to the last 10 years, since the concepts of social robots and user experience evaluation techniques have witnessed progress recently.

### 2.4. Study Selection and Data Collection Process

**Figure 1:**
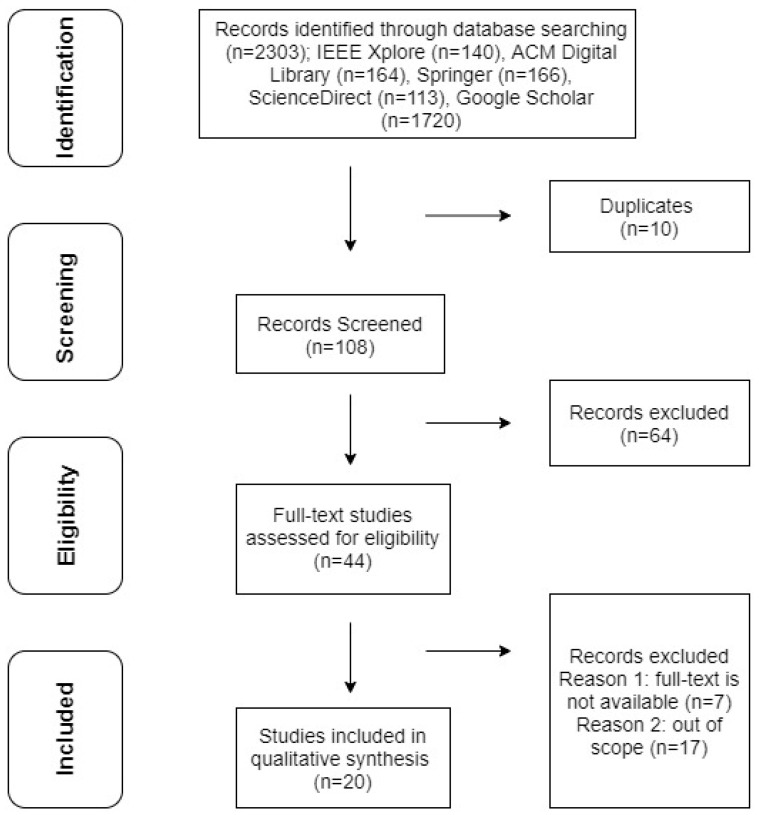
PRISMA flow diagram of study selection process.

The study selection process is represented by means of the PRISMA flow diagram (See Figure 1). A total of 2303 studies (IEEE Xplore (n = 140), ACM Digital Library (n = 164), Springer (n = 166), ScienceDirect (n = 113), Google Scholar (n = 1720)) were identified during the initial search process. The title and metadata of these studies were examined, and 2185 studies were evaluated as unsuitable for this systematic review. A further 10 studies were excluded because of duplication. In addition, 64 studies were excluded as a result of assessing their abstracts against inclusion/exclusion criteria. Consequently, only 44 studies were selected for the eligibility stage. Inclusion/exclusion criteria were applied to the full texts of these studies and only 20 studies were finally included in this systematic review.

### 2.5. Data Extraction and Synthesis

This study used Zotero to extract attributes from the selected studies in an automatic fashion, such as study title, author(s) name(s), publication year, publication source and keywords. In addition, two of the authors extracted the research topic and themes independently. Previous literature has introduced two data synthesis approaches: descriptive/narrative and quantitative data synthesis [66]. This study adopts the descriptive data synthesis approach that encompasses a simple description of topics and techniques in order to answer RQ1 and RQ2, and the identification of themes to answer RQ3. The themes were discussed among three of the authors. Disagreements that emerged due to conflicting themes were resolved with the contribution of the third author who is experienced in HRI research.

### 2.6. Risk of Biases

The studies selected for this review were assessed with regards to the risk of biases. The following types of biases were identified in these studies: (i) sampling biases due to the selection of a homogeneous group of users. For instance, Gerłowska et al. [67] selected elderly with no significant differences in age, gender, education level, and similar attitude towards novel technology as the participants of their study, whereas Reich-Stiebert and Eyssel [68] selected homogeneous university students without considering other groups of interest such as teachers and parents. Cesta et al. [69] adopted non-probability sampling methods: convenience sampling combined with chain sampling methods. This combination of methods has been referred to as mixed purposeful sampling [69]. (ii) measurement biases. In order to investigate the attitude towards education robots, Reich-Stiebert and Eyssel [68] provided a written description of characteristics and functions of education robots without using picture materials of implemented education robots. Destephe et al. [70] aimed to investigate the relevance of attractiveness on the acceptance of a robot as a partner in the working environment. The authors of this study used videos as stimuli, as a useful means to explore indirect interactions. However, it does not enable the understanding of real interactions with users. De Graaf and Ben Allouch [71] investigated variables influencing the acceptance of social robots. Due to the short-term nature of this study, the authors measured a novelty effect. Therefore, they recommended long-term studies to omit such an effect.

## 3. Results

In this section, we address the research questions that were defined in Section 2.1. The full list of primary studies are collected in Appendix A.

### 3.1. RQ_1_: What Has Been Studied About User Experience in Social Robots Operating in Different Domains?

**Figure 2:**
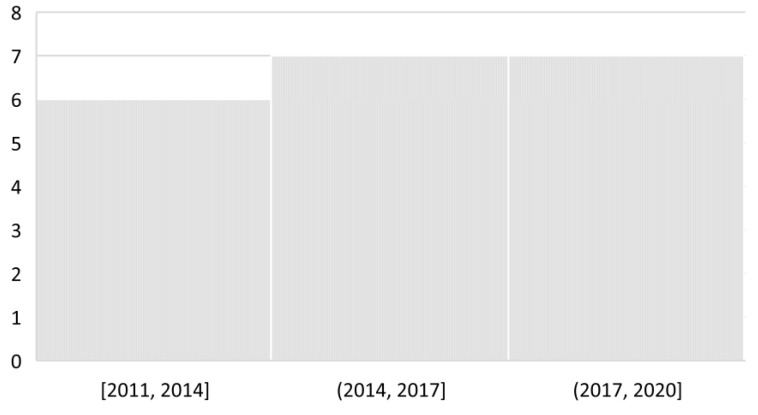
Frequency of studies per period of time.

Prior to answering RQ_1_, it is important to observe the trend of the studies related to the topic of interest. Figure 2 depicts the frequency of studies published per period of time. We observed a slight increase of the number of studies published during the second period of time (2014, 2017] compared to the first period.

For many years, robots have been tools used primarily in factories [72]. Nowadays, robots have become embedded in everyday people’s life and co-operate in both the industrial and service sectors. In particular, social robots are expected to play an important role in human society [41]. However, to achieve long-term benefits to the lives of people, there is a need for an iterative and positive UX when interacting with social robots. Social robots are being used in various application domains such as home use, manufacturing, healthcare, and education. One may argue that considering human-robots interaction in the design of robots can increase their usefulness and safety [41].

One of the social robots’ applications is in the care and assistance domain. Indeed, it is complicated to provide care and assistance services to people who demand special care. Telepresence robots are robots that are being used in different contexts, especially for elderly assistance [69]. Cesta et al. [69] described their experience with using telepresence robots in a real context for a long-term period. Their evaluation indicated that the telepresence robot was perceived positively by both males and females in terms of perceived usefulness, intention to use and attitude. These findings rely on the MARTA (**M**ultidimensional **A**ssessment of telepresence **R**obo**T** for older **A**dults) methodology, which defines a set of variables of interest in the interaction of telepresence robots and users. Furthermore, the results revealed that the perceived usefulness, intention to use and perceived adaptiveness of users towards telepresence robots increased over time. In addition, primary users stated that they would be willing to continue using the robot showing a positive attitude towards a telepresence robot, namely Giraff [69].

Gerłowska et al. [67] assessed the impact of assistant robots on aging patients with memory impairments. The authors conducted experiments on participants (55–90 years old, both genders) with and without cognitive impairments. The participants were asked to carry out some daily tasks like cooking, leisure time, medication intake, and social interaction. In order to support the participants while performing the tasks, RAMCIP (Robotic Assistant for Mild Cognitive Impairments Patients at Home) was used. The authors conducted an in-depth analysis of the impact and attractiveness of the robots, and the results revealed that the assistant robot was highly assessed and easy to get familiar with. On the other hand, usability was considered neutral. However, the authors reported that they needed a longer interaction to grasp it properly. Additionally, for the matter of societal impact, the robot is recognized as highly advantageous for patients’ health and wellbeing [67].

Destephe et al. [70] aimed to enhance the understanding of the Uncanny valley phenomenon, i.e., humanoid robots may cause uneasy feeling in human observers. To achieve this goal, the authors investigated factors that could have an impact on our perception of humanoid robots. They carried out a cross-cultural study using videos based on changing the motions of a humanoid robot, namely WABIAN-2R and distributed questionnaires to 69 subjects. Their findings suggested that the main influencing factor of the Uncanny valley feeling is the attitude towards humanoid robots. Subjects with a positive viewpoint regarding robots rated the robot as less eerie and more attractive than subjects with negative viewpoint. In turn, the perceived attractiveness of the robots impacts in a significant manner its occupation acceptability regardless of the perceived eeriness.

Humanoid robots may also be applied in other domains such as learning and teaching processes. In fact, in many countries, they are being used as guidance in science classes. According to a survey conducted among German university students, most of the participants prefer education robots as co-teachers and assistants in the classroom and just a miniature part of them perceive humanoid robots as independent teachers. Overall, the results of the survey showed a relative reluctance to involve robots in the learning processes [68].

Šabanović [73] carried out observational work in Japan, with the goal to explore how roboticists design social robots. Robotics in Japan are easily accepted by society as social agents. In order to advance robotics, scientists proposed the incorporation of traditional themes and cultural values with robotics to promote cultural continuity. Using cultural frameworks for novel robotic technologies facilitates the understanding among roboticists that robotics need to not only fit into, but also should be supported by suitable cultural structures.

Using social robots for commercial applications is another scope of robotics operation. Accordingly, Tonkin et al. [74] applied a human-robot interaction (HRI) methodology to the trial implementation of a social robot at an airport. They integrated the Lean UX with HRI research to design robots more efficiently. According to Gothelf et al. [75], Lean UX refers to the evolution of the product design and team collaboration. Essentially, it is an integration between the best part of the designer’s toolkit with agile software development and lean startup thinking, which is available to the entire product team. Tonkin et al. [74] tested the robot at the airport check-in and gate environments for one week. The results revealed that more than 50% of the users interacted with the robot via touchscreen or voice to request a joke. However, they rarely had a voice interaction. Besides, checking carry-on baggage size by the robot was not accepted by any user [74].

Table 1 provides information about the studies used in this section to answer RQ_1_. We selected a subset of the primary studies (8 out of 20, 40%) which explicitly discussed the perception of users towards social robots operating in different areas.

### 3.2. RQ_2_: What Are the Reported Techniques for Assessing UX in Social Robots?

There are many factors that determine the proper UX evaluation method including but not limited to, timespan, purpose, field, financial resources, supply, and methodological knowledge [44]. The received feedback from users can be assessed and measured in two ways: formative and summative [44]. Formative evaluation aims at getting feedback on conceptual design ideas during the development process by using techniques such as interaction flows, rough sketches of the design of robots and physical mock-ups. On the other hand, summative evaluation aims at understanding the robots’ usage in its real context. Therefore, the focus is on evaluating the final robot. However, it is more complicated, time-consuming, and expensive to change the interaction design and robot’s interface in the latter development stages. Thus, it is recommended to perform formative and summative evaluations throughout the whole design process [44]. In order to have a better approach to answer the questions “what to do” and “when one should use which method”, the following chart illustrates the procedure and method selection of the UX evaluation. This is a general method to evaluate user experience in each specific context. The vertical axis demonstrates the attitudinal vs behavioral dimensions, and the horizontal axis refers to qualitative vs quantitative evaluation. Each dimension determines the most suitable way of evaluation and types of questions that can be asked for the intended purpose [44,76].

To have a successful UX evaluation, the robot developer or UX researcher should focus on facets like picking the UX aspects and methods, data collection method, and target that for the specific area [77]. The UX evaluation involves a wide range of techniques, including empirical methods such as lab-based evaluation and analytical methods [44,77]. While it is true that the use of all methods in every HRI project is not feasible, it is also true that the use of several methods leads to a better UX evaluation, e.g., not only conducting contrived experiments including questionnaires [78].

Greunen [41] advocates the importance of UX evaluation, although investigations are often carried out after the actual human-robot interaction. Consequently, the subjects reflect upon their interaction with the social robot afterwards, and this might introduce biases, which can affect the reliability of the investigation.

As indicated by many researchers, robots in real environments demand long-term evaluation studies. Moreover, it is necessary to involve people in the design process for developing robots by having a real experience in their homes during their daily life. The methodology for collecting data in participants’ homes can be fulfilled by questionnaires, interviews, sensors, and robot logs [79,80]. After designing the robot, the evaluations are often performed in laboratories due to insufficient time without considering the prolonged real-world challenges [69].

Regarding long-term evaluation, it is necessary to gather data from human-robot interaction and user experience during a specific period. To collect these data, there are different methods and techniques. For instance, direct observation and recorded observation are primary observational techniques in which the researcher directly observes the interaction between the user and the robot, monitors and documents the observations. In recorded observation, the researcher is not present in the session, and the observations are captured by means of video recording. Each technique is suitable for a certain purpose. For instance, if the focused interaction and behavioral aspects are clear from the beginning, direct observation is suitable because it is easy to analyze that amount of data. Recording sessions and going through the records in order to collect data afterwards is a time-consuming process. Nonetheless, if any features should be measured in detail, recorded observation is the recommended approach [42,44].

Cesta et al. [69] present the MARTA methodology. This methodology denotes the variables that are principal for the evaluation of the adoption and effective use of social robots over time in order to investigate the consequences of habituation and potential reasons for rejection by users. In this assessment, the authors involved quantitative and qualitative instruments (questionnaires and interviews/diaries) to reveal users’ needs properly [69]. Interview is a method in which the researchers can gain profound information about users’ feelings and their way of thinking. Furthermore, questionnaire is a quantitative data collection technique for gathering subjective data about how users view the design [42].

Gerłowska et al. [67] targeted the assessment of RAMCIP in a semi-controlled environment. The assessment centered on the functionality, usability, level of the perceived acceptability, and societal impact by the end-users. The authors evaluated the functionality of the RAMCIP by means of User Experience Questionnaire (UEQ) and a survey to assess societal impact. Furthermore, in order to assess the attractiveness and acceptance, the authors carried out a User Experience Questionnaire in which the measurements clustered in six scales [67]. The UEQ scales involves [81,82]:

- Attractiveness: the impression of the product
- Perspicuity: easy to use and follow the product
- Efficiency: solving users’ task without additional work
- Dependability: users’ feeling of being in control of the interaction
- Stimulation: how engaging the product is
- Novelty: the novelty of the product

Destephe et al. [70] conducted a study on the acceptance of the robot as a working partner and factors that might affect the perception towards the robot. The authors conducted an experiment to detect where the participants would see the robot carrying out a task. To accomplish this assessment, the authors distributed questionnaires to the participants. The questionnaires involved inquiries about general information of participants, their robot-related experiences, their attitude towards robots based on another questionnaire called MacDorman, personality questionnaire, and participants’ reactions and feelings about robots based on Ho’s questionnaire. The questionnaire intended to measure three categories [70]:

- Perceived Humanness: the level of humanity and human-like characteristics of the robot
- Eeriness: the feeling of strangeness, disgust, and familiarity
- Attractiveness: the level of physical attraction

Accordingly, the results showed that attractiveness is the main factor in predicting occupation acceptability.

Sabanovic et al. [79] conducted in situ evaluation to investigate multiple design alternatives of the break management robot, one that functioned as a simple alarm and the other with social behavior. Before the experiment, participants enrolled in semi-structured interviews and online questionnaires to be questioned about their break-taking, work practices, and attitudes towards technology. The questionnaires were inspired by the “Technology Attitude Instrument”, “Perceived Usefulness” scale, full “Negative Attitudes Towards Robots Scale” (NARS), and “Emotional Contagion Scale”. The user experience was evaluated over four consecutive weeks. Half of the participants were given the socially interactive prototype and the others received the alarm to use for two weeks. The participants described their first-week experience through a self-report. Throughout the second and third week, behavioral self-reports from users and logs recorded by the robots were collected. At the end of the experiment, the participants were asked to fill the “Adoption of Information Technology in the Workplace” online survey, which included the voluntariness of future use, perceived relative advantage, compatibility, image, and ease of use [79].

Table 2 presents an example of a questionnaire that is called NARS. The NARS questionnaire has scales based on participants’ responses to explain differences in their behavior and tensions regarding the interaction with robots. The grades of the answers are 1: Strongly disagree, 2: Disagree, 3: Neutral, 4: Agree, 5: Strongly agree. Then, the scores of all the items are added up with the reverse of scores in some items. By adding up the scores of all the items included in the subscale, with the reverse of scores in some items, the individual’s score can be measured. Hence, the minimum and maximum scores are 6 and 30 in S1, 5 and 25 in S2, and 3 and 15 in S3, respectively [83].

The NARS items are classified into three subscales which can be described as follows:

- Sub-scale 1: Negative Attitudes towards Situations and Interactions with Robots (six items)
- Sub-scale 2: Negative Attitudes towards Social Influence of Robots (five items)
- Sub-scale 3: Negative Attitudes towards Emotions in Interaction with Robots (three items)

The analysis of these questions guided researchers to investigate the attitude and experience of users towards robots. Moreover, in order to have a more natural HRI, empathy plays a crucial role in interacting with social robots [84]. Ficocelli et al. [85] performed an emotion-based assistive behavior in social assistive robots, and from users’ point of view, it improved robots with more proper emotions.

Table 3 summarizes the user experience evaluation methods which are discussed in this section. It is noteworthy that Table 4 includes only a subset of the primary studies because these studies present the user experience evaluation method explicitly.

Overall, most of the UX of SR assessments are performed by means of interviews, surveys, and daily self-reporting. Other evaluation methods do exist, such as lab-based studies, direct observation, and recorded observation which are becoming popular in HRI studies. These methods have been considered as fair approaches because the users are not able to consciously manipulate the activities and procedures. However, in some cases, these methods have been refused due to the participants’ wishes [79,86].

### 3.3. RQ_3_: What Are the Reported Challenges and Benefits in Evaluating UX for Social Robots?

In this section, the authors identify and discuss benefits and challenges related to evaluating UX of human-robot interaction. The authors outline the value of receiving feedback from users in early stages of the development process, and the value of setting UX goals for robot developers. On the other hand, the UX evaluation of HRI faces challenges related to defining and incorporating UX goals, challenges related to the users’ first experience with social robots, and the limited number of studies that consider UX evaluation of HRI in its real-world context. In what follows, these benefits and challenges are discussed.

Benefits in early-stage feedback: In any interactive systems including social robots, a positive UX is inevitable to harvest the expected privileges. To achieve a positive UX, Lindblom et al. [44] encourage the inclusion of formative evaluations throughout the entire design lifecycle process. Formative evaluations entail receiving feedback on conceptual design ideas in the early stages of the UX design process. This initial feedback from users provides valuable information on the interaction quality, choosing among multiple alternative designs, recognizing UX obstacles and a negative UX. The earlier identification of these obstacles leads to easier, less time-consuming and more financially viable modifications of the robots’ design or interaction flow than in the latter stages.

Benefits for robot developers: UX goals foster robot developers to focus on the expected experience of interacting with robots. Therefore, the evaluation process has the potential to clarify what exactly should be done to improve specific aspects of the robots’ UX. During the design process, UX goals set some quantitative and qualitative metrics which assist robot developers in understanding when the required quality of interaction has been achieved, when to stop repeating the design process and when it is deemed to achieve a successful design [42,44].

Challenges of defining relevant UX goals: UX goals can be defined as high-level objectives or desired effects that must identify aspects that are important to the user when interacting with a specific system [87]. As recently pointed out by Lindblom and Andreasson [87], although defining UX goals is a fundamental activity, it has been overlooked in HRI, probably due to the lack of knowledge or time. In fact, these UX goals may serve as evaluation metrics, which support the UX evaluation process and enable developers to reflect upon the evaluation outcomes. It is worthy to note that UX goals focus on the human-robot interaction quality rather than the evaluations of the robots’ behavior and functionalities. Therefore, these goals support the whole development lifecycle by identifying when the desired interaction quality has been achieved.

Challenges of the first User Experience: People may feel unnatural during their first experience with social robots, but they may find it useful and well-adapted after a more prolonged time of interaction. Yet, some people may find it boring, and some find it interesting. Hence, to design the robot with the expected user experience, it is crucial to recognize what kinds of feelings a robot should arouse, and at what level the robot is expected to evoke the intended user experience [87]. These are the aspects of HRI that should be evaluated by means of long-term studies, given that human-robot interactions may vary from trial to trial, due to the autonomous nature of social robots [87].

Challenges of limited assessment: It is important to highlight that human interaction with SR has been assessed only to a limited extent because it is often accomplished in laboratories for a short period of time. The assessment outcomes are solely based on the results of some pre-produced tasks that a limited number of users are given. Thereby, these assessments overlook real-world challenges, and consequently constrain the expansion of social robots beyond laboratory settings. Likewise, a similar conclusion was reached by Dautenhahn [78], who outlined the need for HRI studies to explore the long-term interaction between humans and complex social robots in the real-world context and circumstances. Indeed, such studies are complex to design and execute, intensive from a research perspective and time-consuming [78].

Table 4 presents the studies used to answer RQ_3_. These studies discussed the benefits and challenges of the UX evaluation for SR.

## 4. Discussion

### 4.1. UX in Social Robots: An Overview

The fusion of social robots in human life is undeniable, and their importance in human society is increasing [84]. Social robots have been applied in several domains including healthcare [88], education [89], business [90], and culture [91]. The user experience and acceptance of such robots varies in each domain and in different settings (e.g., after a brief initial interaction, as underlined in [92]). For instance, in healthcare, robots were perceived as having positive effects on patient care and communication. Interestingly, assistive robots were well-accepted by elderly people, but were not well-accepted by some care professionals or in the education domain. While some respondents in primary studies believed that robots could participate as a teacher or teacher assistant in subjects such as science and mathematics, others were reluctant to participate in teaching provided by a robot.

A negative user experience may lead to unfortunate consequences such as negative credit or reluctance to use that specific robot [41]. For social robots to bring a long-term value to humans, a positive UX is a requirement. However, it does not occur automatically. Achieving a positive user experience requires an intelligent and systematic design and iterative evaluation [77]. UX evaluation provides an overview of each step of the design and development process and assists in handling the possible errors or imperfections at an early stage. There are various methods to evaluate UX in robotics including questionnaire, survey, interview, self-report, focus group, direct observation, and recorded observation. According to our selected studies, questionnaires and interviews are the most common methods for evaluating UX in social robots. Interviews provide richer information about users’ expectations, judgments, and perceptions than questionnaires. On the other hand, questionnaires render the possibility to gather more quantifiable information such as rating dimensions of UX. It is worth mentioning that, in some cases, the users refused the direct observation and recorded observation methods.

This is in line with the findings of the applicability of questionnaires in UX evaluation e.g., [93,94,95]. Therefore, the findings of this study (See Table 4) provide evidence that the techniques used for UX evaluation in social robots are similar to traditional UX evaluation techniques. While it is true that the adoption of existing practices, techniques and methods from other fields such as UX and human-computer interaction has been encouraged in the extant literature [87], it is also true that there is a need to tailor these practices to the HRI field. As stated by Dautenhahn [78], the field of HRI differs from human-human interaction, human-computer interaction, traditional robotics and engineering research. These differences should be considered when selecting and customizing appropriate UX evaluation techniques.

With regards to the challenges, our studies revealed that setting UX goals is often neglected by robot developers due to the lack of knowledge or lack of time. In fact, the use of an inappropriate strategy leads to a restricted UX evaluation. Hence, robot developers need to obtain the required knowledge in both theory and practice and understand how to perform a successful UX evaluation. This is in line with relevant and recent contributions in the topic [96,97].

### 4.2. Limitations

We carried out this SLR with rigor and guided by sound research methods and guidelines. However, the study faces a set of limitations which are referred to as threats to validity [98]. In what follows, the main threats to validity are explained and justified according to actions taken to mitigate them adapting actions and definition from [99].

Internal validity refers to the ability to make assumptions about the relationship of the study results and reaching the conclusion from causes and effects. The selection of five databases is a threat to internal validity because it may be influenced to some extent by researchers’ biases. However, the databases were chosen based on their popularity. Therefore, we believe that they cover the majority of relevant studies. Furthermore, the development of the field can be another threat to internal validity. We decided to conduct a systematic literature review because the topic of UX in social robots has been studied for a relatively long time.

Furthermore, external validity plays a crucial role in research. External validity concerns the validity of applying the conclusions that can be generalized to other contexts. It is worthy to note that, the selected studies are not restricted to a specific domain, and the benefits and challenges identified are of general nature.

Construct validity refers to the degree to which a test measures what it claims to measure. In our study, the term “user experience” was also mentioned as “UX” in some contexts. Therefore, these terms were included in the search string. Moreover, we recognized that the term “social robots” was also referred to other terms such as humanoid robots. Trial searches were carried out with this term, however additional relevant studies were not identified. Other terms may exist, and this is a potential threat to construct validity. Despite the fact that this study included only a subset of the extant literature, we believe it covers the most relevant primary studies regarding user experience in social robots. Furthermore, this study can present a selection bias due to the academic search engines selected. However, these databases are commonly used in published SLRs and their selection is also based on Kitchenham and Charters [66].

Conclusion validity refers to the reliability of the conclusions. We assured reliability by performing discussion sessions and analyzing our findings. Furthermore, we carried out searches separately and retrieved data about the selected studies. The studies were assessed by two of the authors according to the formulated inclusion/exclusion criteria, and two other authors evaluated the whole process. After brainstorming sessions and negotiations, the final set of studies was selected. To avoid possible human errors regarding the data analysis process, the results were assessed thoroughly by the third author who is experienced in SLRs in this topic.

Finally, to safeguard the replicability of the study, the data used in this SLR can be accessed online as indicated in the Data Availability Statement.

## 5. Conclusions and Future Work

This study focused on user experience in social robots, the benefits of evaluating user experience when interacting with social robots and the challenges that robots developers or UX researchers may face during the assessments. The goal of the study is to summarize the extant literature in this domain, by carrying out a systematic literature review which follows PRISMA guidelines for systematic literature reviews. The formulated search string was applied to a set of databases mentioned in the methodology section in order to extract the relevant results. The initial results were assessed against inclusion and exclusion criteria, and as a result, 20 papers were selected as relevant for this study. It is important to note that this is just a small portion of the existing studies about this topic. Data was extracted from these studies and the results were analyzed and interpreted in order to answer the following research questions: (i) What has been studied about user experience in social robots operating in different domains? (ii) What are the reported techniques for assessing UX in social robots? (iii) What are the reported challenges and benefits in evaluating UX for social robots?

As discussed in this study, for socially interactive robots, it is of major importance to stress the need for positive user experience. Assessing the UX in the early stages of social robots development benefits the process with immediate action to perform modifications, shift to alternative designs, and, consequently, it saves time and expenses. There are several methods to evaluate UX in SR. The best methods can be selected according to the purpose and the UX goals can lead the process to the expected quality and direction.

Based on the findings of this study, the authors suggest further research efforts in four main dimensions, as follows: (i) the interdisciplinary perspective of the interaction between humans and social robots. This entails adopting concepts, methods and practices from more mature fields, such as human factors, experimental psychology, human-computer interaction, anthropology, and ethology, and tailoring them to the field of social robotics; (ii) guidelines to support robot developers on how to define UX goals in terms of which UX dimensions to consider for specific purposes, user needs, usage context and domain; (iii) the use of a more diverse set of techniques to assess UX in social robots, beyond the conventional techniques of questionnaires and interviews. In fact, these techniques provide after-the-fact insights about interaction quality, which may result in biases that inevitably affect the reliability and conclusion validity of the study; (iv) there is a need for naturalistic field studies for the long-term evaluation of UX in social robots. Indeed, the transfer of outcomes from experimental settings to real-world environments is not trivial. While the authors of this study are aware of the complexities of such studies, they strongly believe in the importance of these evaluations for the further expansion of social robots, beyond laboratory settings.

## Figures and Tables

**Table 1:** Studies used to answer RQ_1_.

Study	Publication Year	Author	Scope
[41]	2019	D. v Greunen	Social Robots Application Areas
[69]	2016	A. Cesta et al.	Evaluation of Telepresence Social Robot Assistant for the Elderly
[67]	2018	J. Gerłowska et al.	Assessment of Impact of a Robotic Assistant for Aging Patients with Memory Impairments
[70]	2015	M. Destephe et al.	Assessment of UX the acceptance of a robot as a working partner
[68]	2015	N. Reich-Stiebert et al.	Education Robots and teacher assistance
[73]	2014	S. Šabanović	Integration of Social Robots and cultural and traditional themes
[74]	2018	M. Tonkin et al.	Implementation of Commercial Social Robot at an Airport
[75]	2016	J. Gothelf et al.	Integration of Lean UX with HRI research

**Table 2:** NARS Items with Subscales.

No.	Questionnaire Item	Sub-Scale
1	I would feel uneasy if robots really had emotions.	S2
2	Something bad might happen if robots developed into living beings.	S2
3	I would feel relaxed talking with robots *	S3
4	I would feel uneasy if I was given a job where I had to use robots.	S1
5	If robots had emotions, I would be able to make friends with them. *	S3
6	I feel comforted being with robots that have emotions. *	S3
7	The word “robot” means nothing to me.	S1
8	I would feel nervous operating a robot in front of other people.	S1
9	I would hate the idea that robots or artificial intelligences were making judgements about things.	S1
10	I would feel very nervous just standing in front of a robot.	S1
11	I feel that if I depend on robots too much, something bad might happen.	S2
12	I would feel paranoid talking with a robot.	S1
13	I am concerned that robots would be a bad influence on children.	S2
14	I feel that in the future, society will be dominated by robots.	S2

* Reversed item.

**Table 3:** UX evaluation method used in each study.

Study	Publication Year	Author(s)	Method
[69]	2016	Cesta et al.	Multidimensional Assessment of Telepresence Robot (MARTA)
[67]	2018	Gerłowska et al.	User Experience Questionnaire (UEQ) and survey
[70]	2015	Destephe et al.	MacDorman questionnaire, personality questionnaire and, Ho’s questionnaire
[73]	2014	Sabanovic et al.	in situ evaluation (pre- and post-interview, online questionnaire, self-report, final focus group)

**Table 4:** Studies used to answer RQ_3_.

	Benefits and Challenges of UX in Social Robots	Studies
Benefits	Early-stage feedback	[42,44,77]
	For developers	[42,44]
Challenges	First UX	[87]
	Relevant UX goals	[87]
	Limited assessment	[69,78]

## Data Availability

Data supporting reported results can be found at https://doi.org/10.6084/m9.figshare.14550870 (accessed on 26 July 2021).

## Appendix A

**Table A1:** Selected studies.

ID	Reference	Year	Title	Source
1	[44]	2020	Evaluating the User Experience of Human–Robot Interaction	Book Chapter (HRI)
2	[41]	2019	User Experience for Social Human-Robot Interactions	Conference (AICAI)
3	[84]	2019	A Survey of Behavioral Models for Social Robots	Journal (Robotics)
4	[67]	2018	Assessment of Perceived Attractiveness, Usability, and Societal Impact of a Multimodal Robotic Assistant for Aging Patients with Memory Impairments	Journal (Front. Neurol)
5	[74]	2018	Design Methodology for the UX of HRI: A Field Study of a Commercial Social Robot at an Airport	Conference (HRI)
6	[78]	2018	Some Brief Thoughts on the Past and Future of Human-Robot Interaction	Journal (HRI)
7	[42]	2018	Agile UX Design for a Quality User Experience	Book
8	[81]	2017	Design and Evaluation of a Short Version of the User Experience Questionnaire	Journal (IJIMAI)
9	[69]	2016	Long-Term Evaluation of a Telepresence Robot for the Elderly: Methodology and Ecological Case Study	Journal (Int J of Soc Robotics)
10	[87]	2016	Current Challenges for UX Evaluation of Human-Robot Interaction	Conference (AHFE)
11	[85]	2016	Promoting Interactions Between Humans and Robots Using Robotic Emotional Behavior	Journal (IEEE Trans Cybern.)
12	[70]	2015	Walking in the uncanny valley: importance of the attractiveness on the acceptance of a robot as a working partner	Journal (Front. Psychol.)
13	[68]	2015	Learning with Educational Companion Robots? Toward Attitudes on Education Robots, Predictors of Attitudes, and Application Potentials for Education Robots	Journal (Int J of Soc Robotics)
14	[86]	2015	Extensive assessment and evaluation methodologies on assistive social robots for modelling human–robot interaction	Journal (IS)
15	[73]	2014	Inventing Japan’s ‘robotics culture’: The repeated assembly of science, technology, and culture in social robotics	Journal (Soc Stud Sci)
16	[79]	2014	Designing Robots in the Wild: In situ Prototype Evaluation for a Break Management Robot	Journal (HRI)
17	[80]	2014	Review: Seven Matters of Concern of Social Robots and Older People	Journal (Int J of Soc Robotics)
18	[76]	2014	When to Use Which User-Experience Research Methods	Nielsen Norman Group
19	[71]	2013	Exploring influencing variables for the acceptance of social robots	Journal (RAS)
20	[77]	2011	User Experience and Experience Design	Book Chapter
